# Hepatitis B e Antigen Status and Hepatitis B DNA Levels in Women of Childbearing Age with Chronic Hepatitis B Infection Screening for Clinical Trials

**DOI:** 10.1371/journal.pone.0121632

**Published:** 2015-03-19

**Authors:** Tram T. Tran, Stuart C. Gordon, Scott Fung, Phillip Dinh, Leland Yee, Eduardo Bruno Martins, Maria Buti, Patrick Marcellin

**Affiliations:** 1 Liver Disease and Transplant Center, Cedars-Sinai Medical Center, Los Angeles, California, United States of America; 2 Division of Hepatology, Henry Ford Hospital, Detroit, Michigan, United States of America; 3 Department of Medicine, University of Toronto, Toronto, Canada; 4 Gilead Sciences, Foster City, California, United States of America; 5 Department of Hepatology, Hospital General Universitario Valle Hebron and Ciberehd del Instituto Carlos III, Barcelona, Spain; 6 Department of Hepatologie, University of Paris, Paris, France; UCL Institute of Child Health, University College London, UNITED KINGDOM

## Abstract

**Background:**

Perinatal or mother-to-child transmission of hepatitis B virus (HBV) results in a high frequency of chronic infection. Risk of mother-to-child transmission is associated with maternal viral factors including hepatitis B e antigen (HBeAg) positivity and viral load.

**Aim:**

To investigate associations between age, HBeAg status, HBV DNA levels and genotype in female patients screened for inclusion into two contemporary, randomized HBV trials.

**Methods:**

Retrospective analyses focused on differences between women of childbearing age (≤44 years) and older women. Female patients (N = 355; 18–69 years) were included in the analysis: 41.7% of patients were Asian. In total, 44.4% were HBeAg-positive.

**Results:**

Significantly more women aged ≤44 years were HBeAg-positive compared to women ≥45 years (57.2% versus 27.5%, respectively, p<0.0001), this proportion declined with increasing age. Younger women were significantly more likely to have high HBV viral load (HBV DNA>10^8^ copies mL: ≤44 years 46.0% vs ≥45 years 25.5%, respectively; p<0.0001), and this declined with increasing age. HBeAg positivity was slightly higher in Asian women, associated with a higher proportion of HBV genotypes B and C in this population. There was no obvious relationship between genotype and viral load.

**Conclusions:**

Women of childbearing age with CHB are more likely to have high HBV viral load and HBeAg positivity than older women; this likelihood decreases with age. Maternal serological and virological status should therefore be established early in pregnancy, taking into account age and genotype, and a risk-reducing strategy implemented in any patient who is HBeAg positive and has a high viral load.

## Introduction

Recent estimates from the World Health Organization indicate that more than 240 million people are chronically infected with hepatitis B virus (HBV), and therefore at risk of serious liver disease, including cirrhosis and hepatocellular carcinoma [1]. The likelihood of developing chronic HBV infection (CHB) depends on age at the time of infection: 80–90% of infants infected during the first year of life and 30–50% of those aged 5 years and under will develop chronic infections compared with fewer than 5% of otherwise healthy adults [1]. Perinatal or mother-to-child transmission (MTCT) results in a high frequency of CHB, especially in highly endemic areas where HBV is still most commonly spread from mother to child. Even in areas of low HBV endemicity, more than a third of CHB cases have been attributed to MTCT or early childhood transmission [1].

Several factors have been identified as increasing the risk of MTCT including maternal hepatitis B e antigen (HBeAg) status. HBeAg positivity in women of childbearing-age is a risk factor for intrauterine infection [2] and, consequently, a major determinant of MTCT [3–5]. The precise mechanisms behind the high rate of infectivity in infants born to HBeAg-positive mothers remain unclear, but may be related to the fact that maternal HBeAg positivity is strongly correlated with high levels of maternal viremia [5–7]. Together with maternal HBeAg positivity, HBV DNA level is an important, if not the most important, predictor and risk factor for MTCT [8–10].

Immunoprophylaxis (both active and passive immunization) against HBV is the mainstay of disease prevention. However, although adoption of standard immunoprophylaxis has reduced the rate of MTCT, up to 8–39% of infants born to highly viremic (defined as HBV DNA >10^6^–10^8^ copies/mL, depending on study) HBeAg-positive mothers still become infected with HBV [5,7,10–14]. High levels of maternal HBV DNA have also been associated with failure of neonatal HBV vaccination (so-called breakthrough infection) and passive-active immunoprophylaxis in infants of HBeAg-positive mothers [2,7,8].

The aim of our study was to investigate the associations between age, HBeAg status, HBV DNA levels and genotype in female patients screened for inclusion into two contemporary large randomized clinical HBV trials, focusing on differences between women of childbearing age (≤44 years) and older women (≥45 years). The data from our study contribute to understanding of the natural history of CHB in women of different ages, and have clinical implications for risk-reduction strategies in pregnancy.

## Materials and Methods

### Study design

This was a retrospective analysis of data from female patients screened for inclusion in one of two phase III studies of tenofovir disoproxil fumarate (TDF) versus adefovir for CHB, that were conducted at 106 clinical sites in 15 countries across North America (USA and Canada), Europe (Bulgaria, Czech Republic, France, Germany, Greece, Italy, Poland, Spain, The Netherlands, Turkey, and UK) and the Asia-Pacific region (Australia and New Zealand).

Patients aged 18–69 years were recruited from May 2005 through to June 2006. Inclusion criteria, the details of which have been previously described [15], included HBeAg-negative status (Study 102; NCT00117676) or HBeAg-positive status (Study 103; NCT00116805) CHB with compensated liver disease together with the presence of hepatitis B surface antigen (HBsAg) for at least 6 months before screening. All screened female patients were included in the current analysis, including those who did not satisfy inclusion criteria for inclusion in the clinical trials. ‘Women of childbearing age’ were defined as those aged between 18 and 44 years (lower limit based on the study inclusion criteria; upper limit based on Centers for Disease Control criteria).

At screening, patients were assessed for the presence of HBeAg, level of circulating HBV DNA and alanine aminotransferase (ALT). HBV DNA was measured using the Roche Cobas Taq-Man polymerase-chain-reaction assay, which has a lower limit of quantitation of 169 copies/mL (29 IU/mL) [15]. The screening assessment also included a complete physical examination and measurement of vital signs. Genotype was assessed from serum samples collected at the baseline visit. Thus, genotypic information was not available for screen failures.

### Ethics statement

The original study from which data were taken was conducted in accordance with international scientific and ethical standards, including but not limited to the International Conference on Harmonization Guidelines for Good Clinical Practice and the Declaration of Helsinki. The study was approved by independent ethics committees or institutional review boards at the study sites. Written informed consent was obtained from all patients before any procedures were performed.

### Statistical analyses

Standard descriptive statistics including mean, median and standard deviation were used to summarize continuous baseline and demographic information; frequency and percentage were used to summarize categorical variables. Comparisons between groups were done via a Wilcoxon Rank Sum test for continuous variables and Fisher’s exact test for categorical variables. Univariate logistic regressions were used to analyze the association between age group and HBeAg status, HBV DNA levels, genotype and race, with age group dichotomized at various levels (≤ 24, 25–34, 35–44, and ≥ 45 years), HBV DNA dichotomized at various levels (> 10^6^, > 10^7^ > 10^8^ > 10^9^ copies/mL), race dichotomized as Asian versus non-Asian. Odds ratios and 95% confidence intervals from these logistic regression models are presented. Associations between HBV genotype and viral load were examined using Fisher’s exact test.

## Results

### Patients

A total of 355 female patients with CHB, of which 197 were HBeAg-negative and 157 HBeAg-positive, were screened for inclusion into the two trials; HBeAg status was unknown in one individual. Overall, 202 patients (56.9%) were aged ≤44 years and were therefore defined as women of childbearing age. Demographic and baseline characteristics of the study population are shown in Table 1.

**Table 1:** Demographic and baseline characteristics.

	Age ≤44 years	Age ≥45 years	Overall	p-value
	(n = 202)	(n = 153)	(n = 355)	
Mean (SD) age, years	31.4 (7.72)	53.4 (5.05)	40.8 (12.81)	<0.0001
Race, n (%)				0.6644
Asian	82 (40.6)	66 (43.1)	148 (41.7)	
Non-Asian	120 (59.4)	87 (56.9)	207 (58.3)	
Geographic region, n (%)				0.3126
Europe	120 (59.4)	79 (51.6)	199 (56.1)	
North America	56 (27.7)	48 (31.4)	104 (29.3)	
Australia/New Zealand	26 (12.9)	26 (17.0)	52 (14.6)	
HBeAg, n (%)				<0.0001
HBeAg-positive	115 (57.2)	42 (27.5)	157 (44.4)	
HBeAg-negative	86 (42.8)	111 (72.5)	197 (55.6)	
Unknown	1		1	
Knodell necroinflammatory score*				0.0903
n	80	65	145	
Mean (SD)	8.0 (2.18)	8.5 (1.77)	8.2 (2.02)	
Knodell fibrosis score, n (%)*				0.0002
0	0	1 (1.5)	1 (0.7)	
1	42 (52.5)	17 (26.2)	59 (40.7)	
2	0	0	0	
3	34 (42.5)	31 (47.7)	65 (44.8)	
4	4 (5.0)	16 (24.6)	20 (13.8)	
Missing	122	88	210	
Mean (SD) HBV DNA, log_10_ copies/mL	7.3 (1.91)	6.7 (1.67)	7.0 (1.83)	0.0002
HBV DNA, copies/mL, n (%)				
>10^6^	146 (72.3)	99 (64.7)	245 (69.0)	0.1334
>10^7^	126 (62.4)	66 (43.1)	192 (54.1)	0.0004
>10^8^	93 (46.0)	39 (25.5)	132 (37.2)	0.0001
>10^9^	42 (20.8)	8 (5.2)	50 (14.1)	<0.0001
Mean HBsAg, IU/mL				<0.001
n	99	68	167	
Mean (SD)	4.2 (0.60)	3.7 (0.62)	4.0 (0.65)	
Median ALT, U/L (range)	69 (8, 627)	69 (16, 806)	69 (8, 806)	0.8375
HBV genotype, n (%)				0.9252
A	14 (14.0)	7 (10.4)	21 (12.6)	
B	13 (13.0)	8 (11.9)	21 (12.6)	
C	23 (23.0)	16 (23.9)	39 (23.4)	
D	47 (47.0)	35 (52.2)	82 (49.1)	
E, F, G, H	3 (3.0)	1 (1.5)	4 (2.4)	
Unknown*	102	86	188	

*Biopsy, HBsAg and genotype were not available for screening failures. ALT, alanine aminotransferase; HBV DNA, hepatitis B viral DNA; HBeAg, hepatitis B e antigen; HBsAg, hepatitis B surface antigen; SD, standard deviation

### Associations between age, HBeAg status, HBV DNA levels and genotype

A comparison of patients in the ≤44 years and ≥45 years cohorts showed that women in the younger cohort were significantly more likely to be HBeAg-positive (p<0.0001) and to have significantly higher mean HBV DNA levels (p = 0.0002) than those aged ≥45 years (Table 1). In addition, a significantly higher proportion of patients ≤44 years had high HBV DNA levels compared with the older cohort (p<0.0001 for both >10^8^ copies/mL and >10^9^ copies/mL HBV DNA cut offs). There were no significant differences between groups with regards to median ALT levels or in genotype distribution. On multivariate analysis, both HBV DNA >10^9^ copies/mL and HBeAg positivity were significantly associated with age ≤44 years (odds ratio (OR) 2.59; 95% confidence interval, 1.12–6.03; p = 0.0267 and OR 2.89; 1.78–4.69; p<0.0001, respectively).

**Fig 1:**
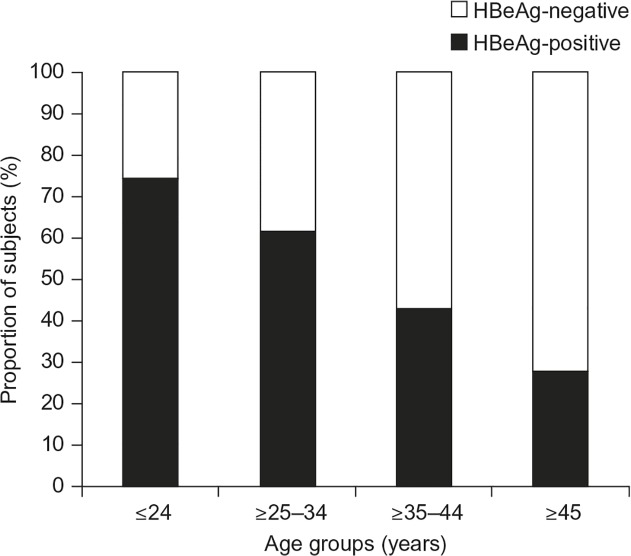
HBeAg status in the four age cohorts.

When divided into four age cohorts (≤24 years, 25–34 years, 35–44 years and ≥45 years), a greater number of younger patients were found to be HBeAg-positive than older patients (Fig. 1). In addition, patients in the younger cohorts (≤44 years) tended to have higher viral loads than older patients, with the highest proportion of patients with HBV DNA levels >10^8^ or >10^9^ copies/mL being in the ≤24 years subgroup (Table 2). The proportion of women who were both HBeAg-positive and who also had a high viral load (and therefore were at highest risk of MTCT) was greater in the subgroups of women aged ≤44 years than in the group of women aged ≥45 years. In the ≤44 years subgroups, the proportion of HBeAg-positive women with high HBV DNA levels using a range of cut-offs was consistently higher in high risk women ≤24 years of age, with the proportion falling with increasing age (Table 3).

**Table 2:** HBV viral load in relation to age*.

HBV DNA levels, copies/mL, n (%)	Age ≤24 years	Age ≥25–34 years	Age ≥35–44 years	Age ≥45 years
	(n = 46)	(n = 82)	(n = 74)	(n = 153)
>10^6^	38 (82.6)	58 (70.7)	50 (67.6)	99 (64.7)
OR (95% CI)	2.59	1.32	1.14	—
	(1.13, 5.95)	(0.74, 2.35)	(0.63, 2.05)	
>10^7^	36 (78.3)	51 (62.2)	39 (52.7)	66 (43.1)
OR (95% CI)	4.75	2.17	1.47	—
	(2.20, 10.25)	(1.25, 3.76)	(0.84, 2.56)	
>10^8^	31 (67.4)	40 (48.8)	22 (29.7)	39 (25.5)
OR (95% CI)	6.04	2.78	1.24	—
	(2.95, 12.36)	(1.58, 4.90)	(0.67, 2.29)	
>10^9^	15 (32.6)	19 (23.2)	8 (10.8)	8 (5.2)
OR (95% CI)	8.77	5.47	2.20	—
	(3.42, 22.49)	(2.27, 13.14)	(0.79, 6.11)	

*Age ≥45 years is the reference category. CI: confidence interval; HBV DNA, hepatitis B viral DNA; OR, odds ratio

**Table 3:** Association of age with high risk of MTCT (HBeAg-positive plus high viral load)*.

HBeAg-positive + HBV DNA levels, copies/mL, n (%)	Age ≤24 years	Age ≥25–34 years	Age ≥35–44 years	Age ≥45 years
	(n = 34)	(n = 50)	(n = 31)	(n = 42)
>10^6^	33 (97.1)	44 (88.0)	26 (83.9)	40 (95.2)
OR (95% CI)	1.65	0.37	0.26	—
	(0.14, 19.01)	(0.07, 1.92)	(0.05, 1.44)	
>10^7^	33 (97.1)	42 (84.0)	25 (80.6)	33 (78.6)
OR (95% CI)	9.00	1.43	1.14	—
	(1.08, 75.05)	(0.50, 4.12)	(0.36, 3.61)	
>10^8^	30 (88.2)	36 (72.0)	18 (58.1)	27 (64.3)
OR (95% CI)	4.17	1.43	0.77	—
	(1.23, 14.10)	(0.59, 3.45)	(0.30, 1.99)	
>10^9^	15 (44.1)	19 (38.0)	7 (22.6)	6 (14.3)
OR (95% CI)	4.74	3.68	1.75	—
	(1.58, 14.20)	(1.31, 10.36)	(0.52, 5.85)	

*Age ≥ 45 years is the reference category. CI, confidence interval; HBV DNA, hepatitis B viral DNA; HBeAg, hepatitis B e antigen; HBsAg, hepatitis B surface antigen; OR, odds ratio

Comparisons between patients of Asian and non-Asian ethnicity showed that ethnic origin had an influence on HBeAg status with rates of positivity slightly higher in Asian women (Table 4). Consistent with observations in the total study population, younger Asian and non-Asian patients were more likely to be HBeAg positive and have high viral loads than their corresponding older cohorts (Table 4).

**Table 4:** Association between ethnicity and age with viral load and HBeAg status

	Asian		Non-Asian		Asian	Non-Asian
	≤44 years (n = 82)	≥45 years (n = 66)	≤44 years (n = 120)	≥45 years (n = 87)	(all ages) (n = 148)	(all ages) (n = 207)
HBeAg-positive, n (%)	60 (74.1)	25 (37.9)	55 (45.8)	17 (19.5)	85 (57.8)	72 (34.8)
OR (95% CI)	4.69 (2.32, 9.46)		3.48 (1.84, 6.61)			
HBV DNA, copies/mL, n (%)						
>10^6^	60 (73.2)	50 (75.8)	86 (71.7)	49 (56.3)	110 (74.3)	135 (65.2)
OR (95% CI)	0.87 (0.41, 1.84)		1.96 (1.10, 3.51)			
>10^7^	54 (65.9)	34 (51.5)	72 (60.0)	32 (36.8)	88 (59.5)	104 (50.2)
OR (95% CI)	1.82 (0.93, 3.53)		2.58 (1.46, 4.55)			
>10^8^	40 (48.8)	19 (28.8)	53 (44.2)	20 (23.0)	59 (39.9)	73 (35.3)
OR (95% CI)	2.36 (1.19, 4.68)		2.65 (1.43, 4.91)			
>10^9^	14 (17.1)	1 (1.5)	28 (23.3)	7 (8.0)	15 (10.1)	35 (16.9)
OR (95% CI)	13.38 (1.71, 104.69)		3.48 (1.44, 8.39)			
HBeAg-positive + HBV DNA, copies/mL, n (%)						
>10^6^	50 (83.3)	25 (100.0)	53 (96.4)	15 (88.2)	75 (88.2)	68 (94.4)
OR (95% CI)	nc		3.53 (0.46, 27.23)			
>10^7^	48 (80.0)	19 (76.0)	52 (94.5)	14 (82.4)	67 (78.8)	66 (91.1)
OR (95% CI)	1.26 (0.41, 3.85)		3.71 (0.67, 20.45)			
>10^8^	38 (63.3)	15 (60.0)	46 (83.6)	12 (70.6)	53 (62.4)	58 (80.6)
OR (95% CI)	1.15 (0.44, 3.00)		2.13 (0.60, 7.54)			
>10^9^	14 (23.3)	1 (4.0)	27 (49.1)	5 (29.4)	15 (17.6)	32 (44.4)
OR (95% CI)	7.30 (0.91, 58.92)		2.31 (0.72, 7.45)			

CI, confidence interval; HBV DNA, hepatitis B viral DNA; HBeAg, hepatitis B e antigen; nc: not calculable; OR, odds ratio

Most patients were infected with genotypes C and D (Table 1). Of those Asian patients with available data (n = 62), with the predominant infections were genotype B (32.3%) and C (56.5%), with few Asian patients infected with genotypes A (4.8%) or D (6.5%). Of non-Asian women with available data (n = 105), the majority were infected with genotypes A (17.1%) and D (74.3%), with few patients having genotype B (1.0%) or C (3.8%) infection (genotypes other than A, B, C or D: 3.8%). Further analysis showed that a higher proportion of genotype B and C infected women (the pattern seen in Asian women) were HBeAg-positive compared with those infected with genotypes A and D (Table 5). There was no obvious relationship between genotype and viral load.

**Table 5:** Association between HBV genotype, HBeAg status and viral load.

	GT A (n = 21)	GT B (n = 21)	GT C (n = 39)	GT D (n = 82)	GT A/D (n = 103)	GT B/C (n = 60)
HBeAg positive, n (%)	10 (47.6)	13 (61.9)	29 (74.4)	27 (32.9)	37 (35.9)	42 (70.0)
HBV DNA, copies/mL, n (%)						
> 10^6^	19 (90.5)	20 (95.2)	34 (87.2)	68 (82.9)	87 (84.5)	54 (90.0)
> 10^7^	15 (71.4)	16 (76.2)	27 (69.2)	53 (64.6)	68 (66.0)	43 (71.7)
> 10^8^	10 (47.6)	12 (57.1)	21 (53.9)	33 (40.2)	43 (41.8)	33 (55.0)
> 10^9^	3 (14.3)	4 (19.1)	6 (15.4)	20 (24.4)	23 (22.3)	10 (16.7)

GT, genotype; HBV DNA, hepatitis B viral DNA; HBeAg, hepatitis B e antigen

## Discussion

This retrospective analysis demonstrates a strong relationship between patients’ age and HBeAg status and viral load. Women of childbearing age (≤ 44 years) appeared significantly more likely to be HBeAg positive and to have high levels of HBV DNA than women aged 45 years and older. The highest proportion of women who were both HBeAg-positive and who had high viral load, and who were therefore at greatest risk of MTCT, was seen in the ≤24 years subgroup in our analysis. Rates of HBeAg positivity were higher in Asian versus non-Asian patients, as would be expected from regional differences in HBV endemicity and the predominance of HBV genotypes B and C in Asian patients which were associated with higher rates of HBeAg positivity in the current study. However, the most striking differences in HBeAg-positive and high viral load prevalence were in relation to patients’ age.

Our finding that younger CHB female patients are more likely to be HBeAg positive and to have high viral loads than older patients is consistent with other studies. In a systematic review, worldwide prevalence of HBeAg positivity in HBsAg-positive women aged 20–39 years was 21–36%, depending on region, with HBeAg positivity rates being lower in those aged over 44 years, and decreasing with increasing age [16]. In the present analysis, 57.2% of women of child-bearing age (≤44 years) were HBeAg positive compared with 27.5% of women aged ≥45 years; in women aged ≤34 years of age in our study, the rate of HBeAg positivity was even higher (65.6%). This is higher than the rates estimated by Ott et al [16], and at the top end of other published prevalence estimates for these age cohorts. For example, in a population-based review, the median seroprevalence estimate of HBeAg in HBsAg-positive pregnant women was 14%, ranging from 0% in the Eastern Mediterranean region to 47.8% in some areas of the Western Pacific Region [17]. However, Ott et al acknowledged that HBeAg positivity rates may have been underestimated in their systematic review. Most studies on HBV seroprevalence included in the review used HBsAg as a marker of infection, but few included information on HBeAg, and several studies that reported HBeAg estimates had to be excluded as they were conducted among high-risk populations or had methodological drawbacks [16]. In addition, it is important to distinguish our study population, which was screened with the intention to participate in clinical trials with strict eligibility criteria (e.g. abnormal ALT levels), from an unselected seroprevalence community-based population that would include, for example, patients with normal and abnormal ALT levels. Our study includes both those patients who fulfilled the study inclusion criteria, and those who failed screening, thus lessening the impact of selection criteria on the findings. Nevertheless the nature of our study population is likely to have contributed to difference between our results and population-based studies.

The finding that younger CHB female patients are more likely to be HBeAg positive and to have high viral loads than older patients is likely a reflection of the natural history of HBV infection. The first immune tolerant phase of infection, characterized by HBeAg positivity and very high levels of HBV DNA, can last from a few years to several decades. It is particularly prolonged where HBV infection is acquired perinatally or in early childhood, the predominant mode of infection in Asians. In such patients the immune tolerant phase can persist into early adulthood, with a low rate of progression to subsequent phases of infection until later life. Where infection arises due to horizontal transmission there is a more rapid progression to the second phase of chronic infection, the HBeAg-positive immune active phase, which is associated with high or fluctuating HBV DNA levels. The timing of further progression to HBeAg-negative CHB, where HBV DNA levels are low, is variable. Younger women are therefore more likely to be in the earlier phases of chronic infection, associated with HBeAg-positivity and high HVB DNA levels. Of all risk factors, a high level of HBV DNA is considered the most important in MTCT [10], with MTCT occurring in cases of high viremia despite active and passive immunoprophylaxis [5–7,18,19]. Data show that risk of MTCT increases significantly in line with increasing HBV DNA levels [7–18]. A number of studies have shown that MTCT is predominantly seen in infants of HBeAg-positive, highly viremic mothers (≥6 log_10_ or ≥7 log_10_ copies/mL) [5–7,18–21], whereas risk is negligible at low viral loads (HBV DNA of ≤4 log_10_ copies/mL) [14]. In the present study, significantly more women aged ≤44 years had high viral loads compared with those aged ≥45 years, which was particularly marked at higher viral load cut-off values (10^8^ copies/mL and 10^9^ copies/mL) and in patients in the youngest cohort (≤24 years of age). This age group was also more likely to be HBeAg-positive with accompanying high viral load and therefore at greatest risk of MTCT.

Treatment with lamivudine and with telbivudine during pregnancy has been shown to reduce serum HBV DNA and HBeAg levels, so decreasing the incidence of perinatal HBV transmission [12–14,22]. TDF, a first-line treatment for patients with CHB and active disease which has been assigned pregnancy category B by the FDA [23], may also be an appropriate option for mothers who may need to continue antiviral therapy for active CHB following delivery. This has been included in a suggested algorithm for risk assessment and prevention of MTCT [10]. This algorithm suggests that, given the high risk of MTCT and high probability of immunoprophylaxis failure, women with high viral load (defined as >10^6^ copies/mL) should receive treatment with lamivudine, telbivudine or TDF (particularly in women with active CHB) in the third trimester of pregnancy [10].

Asian patients in our analysis were predominantly infected with genotypes B and C. We observed no substantial differences in the proportion of patients with high viral loads when stratified by genotype, although infection with genotypes B and C was associated with a higher rate of HBeAg positivity compared with genotypes A and D, with genotype C including the highest proportion of HBeAg-positive women. Evidence regarding the importance of HBV genotype in MTCT is evolving, and existing data are inconclusive. Some studies have failed to demonstrate a link between HBV genotype and risk of MTCT [3,19]. However, higher HBeAg seropositivity rates have been demonstrated for genotype C carriers compared with genotype B carriers [24] and genotype C has been associated with higher rates of infectivity [18,25]. The higher rates of HBeAg positivity in women with genotype B and, especially genotype C infection, and associated increased risk of MTCT, emphasize the importance of MTCT prevention in those parts of the world, such as Asia, where these genotypes are prevalent. In addition, as perinatal infection is associated with an increased risk of chronic infection [1], the higher proportion of HBeAg-positive women, and thus greater potential risk of MTCT, in genotype B and C infection may have implications for the higher prevalence of HBV infection in Asian countries compared with non-Asian countries where other genotypes predominate.

In summary, while it is known that the presence of HBeAg positivity together with a high viral load constitutes a major risk for transmission of HBV from a chronically infected mother to her child, prevalence data are scarce for this high-risk group. In female patients with CHB screened for inclusion into two contemporary, large, randomized clinical HBV trials, women of childbearing age with CHB had a greater likelihood of being HBeAg positive and having a high HBV viral load than those aged 45 years or older. Younger women had the highest risk of being HBeAg positive, and had the highest levels of HBV DNA, a combination which is associated with the greatest risk of MTCT. In addition, the proportion of HBeAg positivity was higher in Asian women in our study compared with non-Asian patients, and in women infected with genotypes B and C, the predominant genotypes in Asian populations. These findings may help to explain higher rate of MTCT in highly endemic areas such as Asia, where age at pregnancy tends to be lower and HBV genotype B and C are predominant.

Our findings have important implications for the management of HBV infection during pregnancy. Asian women (who are predominantly infected with HBV genotypes B and C) may have higher risk of MTCT than non-Asian women, while risk is highest in younger women. It is clear that maternal serological and virological status should be established early in pregnancy, taking into account age and genotype and that a risk-reducing strategy is implemented in any patient who is HBeAg positive and has a high viral load.

(Study 102; NCT00117676, Study 103; NCT00116805)
